# Mode Analysis of Pt/LGS Surface Acoustic Wave Devices

**DOI:** 10.3390/s20247111

**Published:** 2020-12-11

**Authors:** Hongsheng Xu, Hao Jin, Shurong Dong, Xinyu Song, Jinkai Chen, Weipeng Xuan, Shuyi Huang, Lin Shi, Jikui Luo

**Affiliations:** 1 Key Laboratory of Advanced Micro/Nano Electronic Devices & Smart Systems of Zhejiang, College of Information Science and Electronic Engineering, Zhejiang University, Hangzhou 310027, China; iseexuhs@zju.edu.cn (H.X.); dongshurong@zju.edu.cn (S.D.); 21931036@zju.edu.cn (X.S.); 11631009@zju.edu.cn (S.H.); 11731035@zju.edu.cn (L.S.); J.Luo@bolton.ac.uk (J.L.); 2 Hangzhou Global Scientific and Technological Innovation Center, Zhejiang University, Hangzhou 310018, China; xuanweipeng@hdu.edu.cn; 3 Ministry of Education Key Laboratory of RF Circuits and Systems, Hangzhou Dianzi University, Hangzhou 310018, China; chenjk09@hdu.edu.cn

**Keywords:** surface acoustic wave, langasite, mode analysis, platinum, velocity dispersion

## Abstract

Platinum (Pt) gratings on langasite (LGS) substrates are a widely used structures in high temperature surface acoustic wave (SAW) devices. Multiple modes can be excited in Pt/LGS SAW devices owing to the heavy weight of the Pt electrode and leaky waves in the LGS substrate. In this work, we report on a detailed mode analysis of Pt/LGS SAW devices, where three commonly used LGS cuts are considered. A three-dimensional (3D) finite element method (FEM) numerical model was developed, and the simulation and experiment results were compared. The experiment and simulation results showed that there are two modes excited in the Pt/LGS SAW devices with Euler angle (0°, 138.5°, 27°) and (0°, 138.5°, 117°), which are Rayleigh-type SAW and SH-type leaky wave, respectively. Only the Rayleigh-type mode was observed in the Pt/LGS SAW devices with Euler angle (0°, 138.5°, 72°). It was found that the acoustic velocities are dependent on the wavelength, which is attributed to the change of wave penetration depth in interdigital transducers (IDTs) and the velocity dispersion can be modulated by the thickness of the Pt electrode. We also demonstrated that addition of an Al_2_O_3_ passivation layer has no effect on the wave modes, but can increase the resonant frequencies. This paper provides a better understanding of the acoustic modes of Pt/LGS SAW devices, as well as useful guidance for device design. It is believed that the Rayleigh-type SAW and SH-type leaky waves are potentially useful for dual-mode sensing applications in harsh environments, to achieve multi-parameter monitoring or temperature-compensation on a single chip.

## 1. Introduction

In the last several decades, surface acoustic wave (SAW) devices have been explored extensively for sensing applications in harsh environments with great potential [1,2]. The main concerned factors in the development of high temperature SAW devices are the selection of piezoelectric materials and metal electrodes, that determine the stability and lifetime of SAW devices in harsh environments [3,4]. Langasite (LGS) is the material of choice for fabricating high temperature SAW devices, as it offers a relatively high electromechanical coupling coefficient (twice that of quartz) with low propagation attenuation, and most importantly, the ability to operate up to its melting temperature of 1470 °C without phase transition or chemical decomposition [5,6]. As a high temperature material, platinum (Pt) is an attractive material for fabricating electrodes because it can maintain its low resistivity below 748 °C [7]. To allow SAW devices to operate at higher temperatures—up to about 1000 °C or higher—composite metal electrodes or Pt metal with an Al_2_O_3_ passivation layer electrodes have been widely adopted [8,9]. As such, Pt/LGS has been an essential and most important structure of high temperature SAW devices.

In recent years, a large number of studies on Pt/LGS SAW devices have published, and many attractive applications have been explored, among which wireless passive sensing in harsh environments was extensively investigated. Schulz et al. presented the temperature performance of Pt/LGS SAW device up to 800 °C [10,11,12]; Maskay et al. developed a Pt-alloy-based LGS SAW device for strain sensing [13]; Thiele et al. reported on the gas sensing performance of Pt/LGS SAW devices up to 750 °C [14]. In hostile environments such as gas turbines [15], high temperature, high pressure and large strain often coexist, and monitoring these parameters is very crucial for machine state monitoring and structure health maintenance. Based on the application scenarios, Pt/LGS SAW device(s) with more than one acoustic wave mode would be very attractive as multi-mode sensing can be utilized for single chip multi-parameter monitoring or temperature compensation by a differential method [16,17,18]. It was reported that the large mass loading of Pt, nearly eight times heavier than aluminum (Al), could affect the reflection coefficient of LGS SAW devices, and induce multiple modes excitation and interactions between them [19]. However, most reported Pt/LGS SAW sensors only involve a single resonant mode, and detailed mode analysis of Pt/LGS SAW devices is lacking till now. The research by Naumenko et al. revealed the effect of anisotropy on SAW behavior and the interaction of resonant modes of Pt/LGS SAW devices, with Euler angles such as (0°, 22°, 90°) and (0°, 138.5°, 26.7°) [19,20,21]. However, their results were based on a numerical technique and were not compared with experimental results, so we are unable to verify the appropriateness of the analysis. Lots of experimental investigations on LGS SAW devices with Euler angles such as (0°, 138.5°, ψ), (0°, 90°, ψ) and (0°, 22°, ψ) have been conducted [12,22,23,24], however only one mode was investigated in these works. For example [12] studied the high temperature parameters of Pt/LGS SAW devices with an Euler angle (0°, 138.5°, 26°), which only referred to Rayleigh waves. In this paper, we report a detailed mode analysis for the Pt/LGS SAW devices both theoretically and experimentally, and the results are expected to provide a better understanding of the acoustic modes of Pt/LGS SAW device, as well as a guidance for device design. Here, LGS SAW devices with three Euler angles [(0°, 138.5°, 27°), (0°, 138.5°, 117°) and (0°, 138.5°, 72°)] were considered, that are all very attractive for sensing applications.

## 2. Experimental

A two-inch 500 μm thick LGS wafer (The 26th Institute, CETC, Chongqing, China) with crystal cut of (0°, 138.5°, 27°) was selected as piezoelectric material. Since the Pt/LGS SAW devices with Euler angles of (0°, 138.5°, 27°), (0°, 138.5°, 117°) and (0°, 138.5°, 72°) have the same crystal cut but different propagation directions, they were fabricated on one wafer, and hereafter they are named Device A, B and C, respectively.

**Figure 1:**
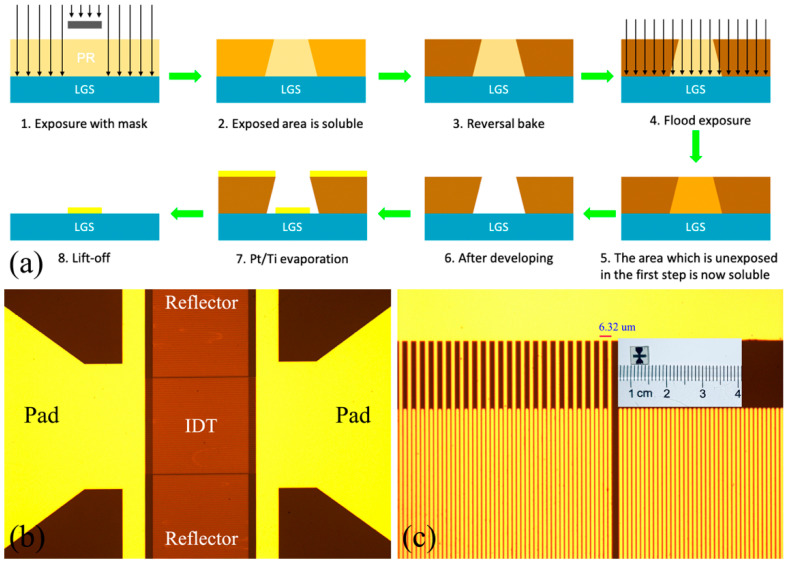
Fabrication steps for the Pt/LGS SAW device (**a**) A micrograph which shows the layout of the device; (**b**) Microscope image of the patterned Pt electrode with a periodicity of 6.32 μm, and the inset is an optical photo of the fabricated device (**c**).

Figure 1a schematically shows the fabrication steps of Pt/LGS SAW devices. The LGS wafer was cleaned with acetone, isopropanol (IPA) and deionized water (DI water) in sequence, and then dried by a nitrogen (N_2_) gun. AZ5214E image reversal resist (AZ Electronic Material Corp., Darmstadt, Germany), which is widely used for lift-off processes [25], was spin-coated @4000 rpm on the surface of LGS wafers with a thickness of 1.4 μm, and the sample was then prebaked at 110 °C for 60 s. UV exposure using projection/stepper lithography (NIKON-I7, Tokyo, Japan) was followed with an intensity of 10 $\mathrm{mW}/{\mathrm{cm}}^{2}$ for 100 ms. To process the image reversal resist as a negative photoresist (PR), a reverse bake at 110 °C for 60 s was applied to the sample, followed by a flood exposure using UV photolithography (365 nm, MA6, SUSS, Garching, Germany) with an intensity of 20 $\mathrm{mW}/{\mathrm{cm}}^{2}$ for 35 s, after which the area that was unexposed in the first step became soluble in developer. Subsequently, the sample was developed using AZ300 MIF developer (TMAH 2.38%, AZ Electronic Material Corp., Germany) for 15 s. To remove the residual resist, the samples were processed by oxygen plasma treatment with a power of 200 W for 2 min. A h=160 nm thick Pt film with a 10 nm titanium (Ti) adhesion layer was deposited on the patterned substrate using an e-Beam evaporation system. To perform lift-off process, the deposited samples were firstly immersed into acetone for 15 min in an ultrasonic bath, then soaked in IPA solution for 15 min in an ultrasonic bath, and subsequently rinsed with DI water for three times, followed by drying using N_2._ After the above procedure, the LGS wafers were successfully patterned with structured Pt electrodes. Then the samples were laser dicing-sawed into SAW devices, prior to which a photoresist layer was spin-coated on the wafers to protect the devices.

Figure 1b is a micrograph of one fabricated Pt/LGS SAW device. The device is a single port resonator, which consists of one interdigital transducer (IDT), two reflectors as well as two pads for wire-bonding. The number of IDT finger pairs is set to 100, the finger number of each reflector is set to 400, and the aperture has a length of 100 λ. Figure 1c is the enlarged view of the Pt metal IDT fingers with a periodicity of 6.32 μm, which shows the well-defined pattern. The inset is an optical photo of the dicing-sawed SAW device with a size of 5 mm × 5 mm.

For testing, the devices were electrically connected to a PCB by wire bonding (WT-2330, WETEL, Shenzhen, China), and a MMCX connector was soldered on the PCB for signal transmission through a coaxial line with an impedance of 50 Ω. The reflection spectrum of SAW devices was measured by a network analyzer (E5071C, Keysight, Santa Rosa, CA, USA).

## 3. Modeling

The surface acoustic waves propagating in a LGS substrate is governed by the following equations [26]:

(1) $$\{\begin{array}{c} c_{ijkl}\frac{\partial^{2}u_{k}}{\partial x_{j}\partial x_{l}}+e_{ijk}\frac{\partial^{2}\phi }{\partial x_{j}\partial x_{k}}-\rho \frac{\partial^{2}u_{i}}{\partial t^{2}}=0\\ e_{jkl}\frac{\partial^{2}u_{k}}{\partial x_{j}\partial x_{l}}-\varepsilon_{jk}\frac{\partial^{2}\phi }{\partial x_{j}\partial x_{k}}=0 \end{array}$$

where i,j,k,l=1, 2, 3, $c_{ijkl}$, $e_{ijk}$, $\varepsilon_{jk}$ are elastic, piezoelectric and dielectric constants respectively, $u_{i}$ the mechanical displacement, ρ the density, ϕ the electrical potential.

A numerical computation program was developed to solve the velocities of LGS with Euler angles of (0°, 138.5°, 27°), (0°, 138.5°, 117°) and (0°, 138.5°, 72°), which is based on the iteration method [27] proposed by Campbell et al. Besides, two-dimensional (2D) and three-dimensional (3D) simulation using COMSOL Multiphysics were also conducted for velocities solving. Plane strain approximation was adopted for the 2D simulation. The LGS material parameters $c_{ijkl}$, $e_{ijk}$, $\varepsilon_{jk}$ and ρ published in literature [28] were used.

Through above three methods, the free ($v_{f}$) and metallized ($v_{m}$) surface velocity of the three LGS cuts were calculated and compared in Table 1.

**Figure 2:**
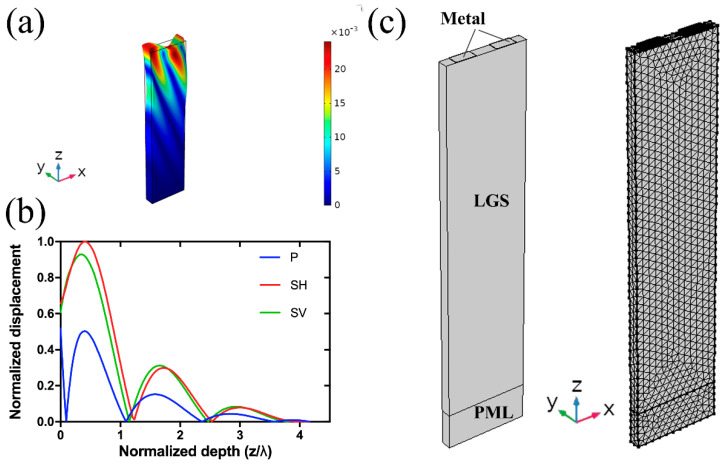
Vibration shape (**a**) and displacement distribution (**b**) of P, SH, and SV wave components of a SAW device (λ=12 μm) based on LGS (0°, 138.5°, 117°); Geometry of the 3D periodic model of the proposed SAW device used in the modeling (**c**).

From Table 1, it is seen that the calculated $v_{f}$ and $v_{m}$ obtained by numerical computation and 3D COMSOL simulation are in excellent agreement, and they also agree well with the reported values [11,29,30]. However, there is a significant discrepancy between the values calculated by 2D COMSOL simulation and by the other two methods. This is because the nonnegligible shear horizontal (SH) displacement component in LGS SAW device cannot be implemented in the 2D COMSOL simulation. The maximum SH displacement for each LGS Euler angle is listed in the last column of Table 1. It is indicated that for LGS (0°, 138.5°, 27°), the calculated parameters by 2D simulation offer the minimum deviation because the SH displacement is the smallest compared to those of other two Euler angles. Giving LGS (0°, 138.5°, 117°) SAW device (wavelength λ=12 μm) with a metallized surface as an example, the total displacement (i.e., vibration shape) and the distribution of acoustic energy are plotted in Figure 2a,b, respectively. Figure 2a clearly shows that there is a leaky wave propagating in the body of LGS substrate, which is due to the existence of SH displacement component [31,32]. In Figure 2b, the displacements of P, SH, and SV waves are plotted as a function of normalized depth (z/λ) into LGS substrate, where P (*x* displacement), SH (*y* displacement), and SV (*z* displacement) represent longitudinal, shear horizontal, and shear vertical wave components, respectively. It was observed that SH and SV waves dominate the majority of acoustic energy, and all the wave components nearly vanish in the depth of 4λ. This is very different from the energy distribution of commonly used 128° Y-cut LiNbO_3_ SAW device, where SH wave is negligible, and the acoustic energy almost decays to zero in the depth of 2λ.

Based on the above analysis, 3D COMSOL simulation was then used to simulate Pt/LGS SAW devices with LGS Euler angles of (0°, 138.5°, 27°), (0°, 138.5°, 117°) and (0°, 138.5°, 72°), namely devices A, B and C. To reduce the computation time and usage of memory resources, one periodic structure with an electrode grating was built, as illustrated in Figure 2c. The element size in *y*-direction of the model is set to 3 μm. As most of the acoustic energy is confined in a depth of 4λ from the surface of LGS substrate, a perfectly matched layer (PML) with a thickness of 10 μm is thus applied to shorten the simulated thickness of LGS substrate to 100 μm, and to suppress wave reflections from the bottom. The left, right, back, and front surface of the geometry are set to a periodic boundary condition. One of the metal electrodes is defined as terminal with 1 V potential, the other electrode is set to ground.

A series of wavelengths are applied so as to cover a wide resonant frequency range from 100 MHz to the 433 MHz ISM-band approximately. We found that the influence of the 10 nm Ti layer on simulation results is negligible, thus the Ti layer is not included in the model. With this FEM/boundary model, it is feasible to obtain the eigenfrequency, mode shapes, and displacement distribution of the simulated structure. The displacements were calculated by setting a series of paralleled cut lines in the *x-z* plane of the model. Equation (2) is adopted in COMSOL to calculate the simulated reflection spectra ($S_{11}$), and plot it together with experimental $S_{11}$, so as to compare the distribution of resonant modes visually:

(2) $$S_{11}=20\;\log_{10}\left(\left(-Y_{11}+0.02\right)/\left(Y_{11}+0.02\right)\right)\;\mathrm{dB}$$

## 4. Results and Discussion

### 4.1. Mode Analysis

**Figure 3:**
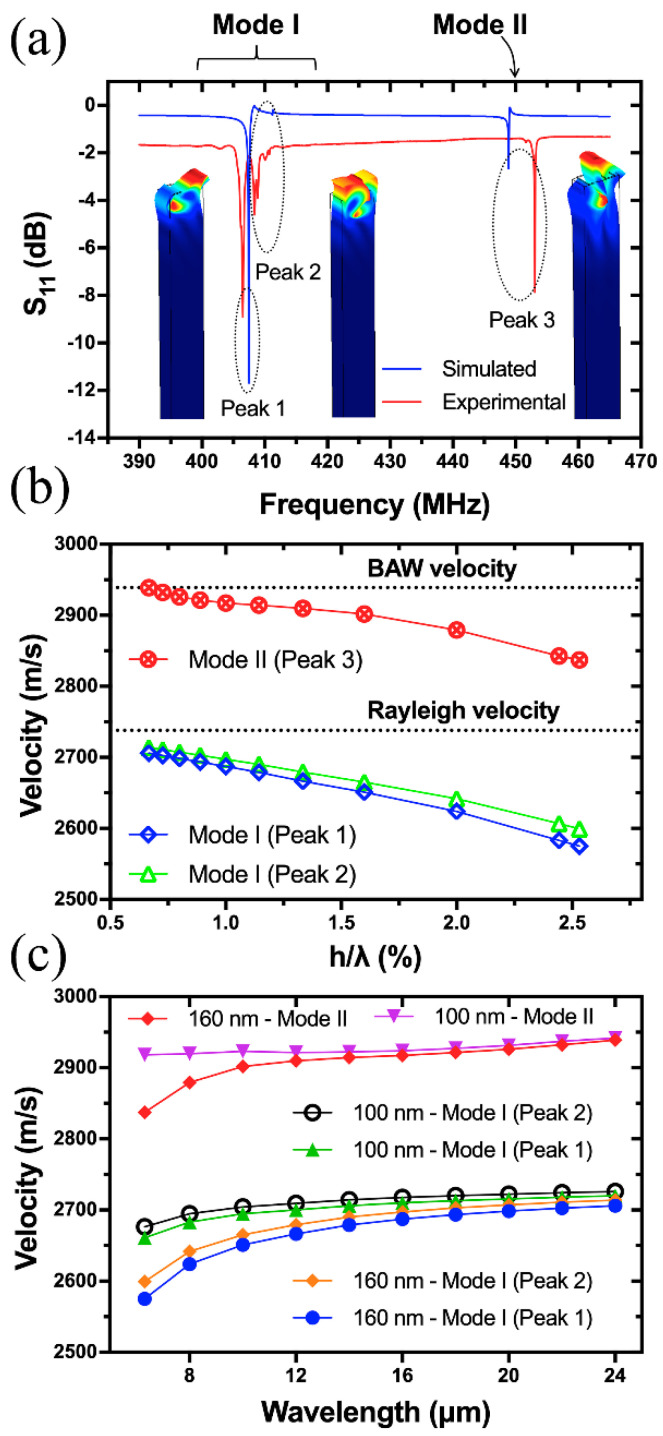
Comparison of the simulated and experimental $S_{11}$ spectra of Device A (λ=6.32 μm) (**a**); Dispersion relations between acoustic velocity and normalized Pt electrode thickness (h/λ) for the two modes of Device A (**b**); Modulation effect of Pt electrode thickness on acoustic velocities of Device A (**c**).

**Figure 4:**
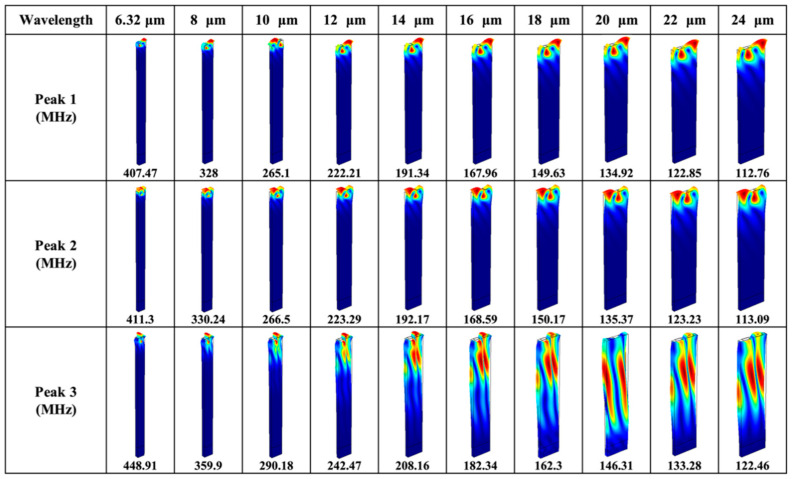
Vibration shapes of Peak 1, 2 and 3 as a function of wavelength in the range from 6.32 to 24 μm of Device A.

Figure 3a is the comparison of simulated and experimental reflection spectra of Device A with a wavelength of 6.32 μm. It is shown that there are two prominent resonance peaks (Peak 1 and 3) and one relatively weak resonance (Peak 2), with frequencies of 407, 450 and 410 MHz, respectively. The small frequency deviations between the theoretical and experimental results are attributed to the nonideal material parameters of LGS substrate used. To clarify the wave types, the resonant shapes and displacement components of the three resonances were analyzed. Figure 4 summarizes the resonant shapes of all the peaks as a function of wavelength in the range from 6.32 to 24 μm, which corresponds to frequency from 112 MHz to that close to 433 MHz ISM-band. It is shown that Peak 1 and 2 are mainly confined on the surface of LGS substrate, and their resonant frequencies are converging as the increase of wavelength. As for Peak 3, its penetration depth into the body of LGS substrate is correlated positively with wavelength.

**Figure 5:**
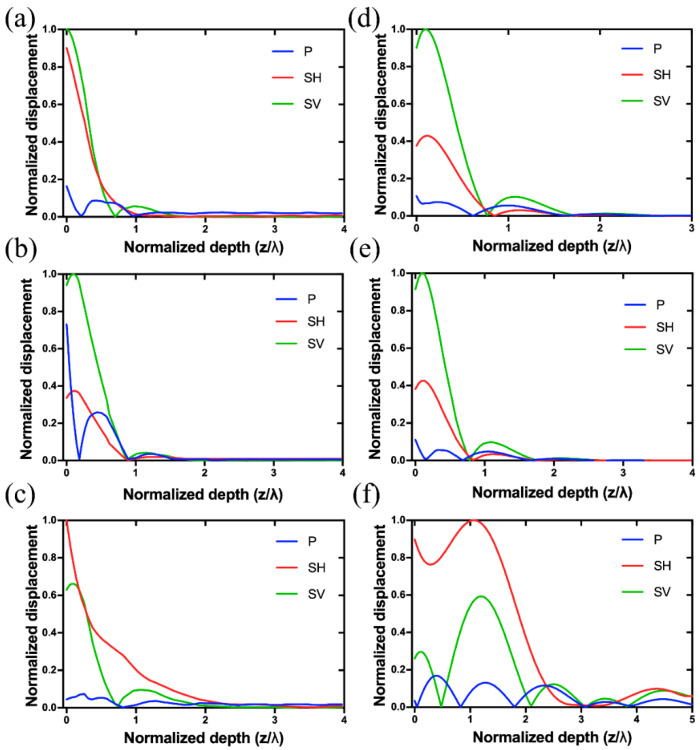
Calculated relative displacements of P, SH, and SV waves of Peak 1 (**a**), Peak 2 (**b**) and Peak 3 (**c**) when λ=6.32 μm; Calculated relative displacements of P, SH, and SV waves of Peak 1 (**d**), Peak 2 (**e**) and Peak 3 (**f**) when λ=20 μm.

For example, most of the acoustic energy of Peak 3 is confined on the surface when λ=6.32 μm (i.e., at ~433 MHz). However, Peak 3 is mainly vibrating inside the body of LGS substrate when λ=20 μm. To quantify this, the displacement components of the three peaks for λ=6.32 μm and 20 μm were analyzed, and the results are shown in Figure 5. Generally, the SV and SH waves dominate the majority of acoustic energy for Peak 1 and 2 when λ=6.32 μm or 20 μm, as shown in Figure 5a,b,d,e. Therefore, it can be interpreted that Peak 1 and Peak 2 have an ellipse polarization rotated around the direction (i.e., x-axis) of wave propagation, which looks like a generalized Rayleigh-type SAW. Also, the acoustic energy of Peak 1 and 2 is mainly confined on the surface of LGS in a depth of λ. Figure 5c,f show that SH wave dominates most of the acoustic energy of Peak 3, and it vibrates in the whole simulated body of LGS with a depth of 5λ when λ=20 μm, which explains the obvious leaky wave indicated in Figure 3a.

The acoustic velocities of all the three peaks as a function of normalized Pt thickness (h/λ) were calculated, where h=160 nm, λ ranges from 6.32–24 μm, and the results are plotted in Figure 3b. The two horizontal lines refer to the Rayleigh ($v_{r}$) and BAW ($v_{b}$) acoustic velocity of LGS (0°, 138.5°, 27°), where $v_{r}$ is the averaged free ($v_{f}$) and metallized ($v_{m}$) surface velocity as calculated in Table 1, $v_{b}$ is slow shear bulk acoustic wave (BAW) velocity. $v_{b}$ can be approximately estimated by the following equation [33]:

(3) $$v_{r}=\frac{0.87+1.12\sigma }{1+\sigma }v_{b}$$

where σ is the Poisson’s ratio of LGS (0°, 138.5°, 27°) with a value of 0.334. Therefore, $v_{b}$ is calculated to be 2939 m/s. The results show that the velocities of Peak 1 and 2 are approaching the Rayleigh velocity (no dispersion takes place) of LGS (0°, 138.5°, 27°) as the decrease of h/λ, and they are nearly coupled with each other when $\frac{h}{\lambda }<0.67\%$ (i.e., λ>24 μm for h=160 nm). Therefore, it is believed that Peak 1 and 2 are both the Rayleigh-type SAWs, and they are the stopband edges of the Rayleigh mode (named as Mode I hereafter) [19,20]. With the decrease of h/λ, the acoustic velocity of Peak 3 is approaching the BAW velocity, and equals to $v_{b}$ when λ=24 μm. Hence, it can be deduced from Figure 3, Figure 4 and Figure 5 that Peak 3 is a SH-type leaky wave (named as Mode II hereafter).

A strong dispersion relation between the acoustic velocity and normalized Pt thickness for both the modes was observed, which is due to mass loading effect. The velocity variations (Δv) of Mode I and II are about −131 m/s (4.8% relatively) and −102 m/s (3.5% relatively) when λ decreases from 24 to 6.32 μm. This is because that, with the decrease of wavelength, the acoustic wave is gradually confined on the surface of LGS substrate, as shown in Figure 4 and Figure 5. Hence, the wave penetration depth into Pt electrodes increases [34], with more acoustic energy coupled into electrodes. As such, Pt electrode plays a role as mechanical resonator, which slows down the acoustic velocity of the device [35]. It can thus be deduced that the acoustic velocity of Pt ($v_{\mathrm{shear}}=1700\;m/s$, $v_{\mathrm{longitudinal}}=3300\;m/s$) is lower than that ($v_{f}=2743\;m/s$) of LGS (0°, 138.5°, 27°) in Device A.

**Figure 6:**
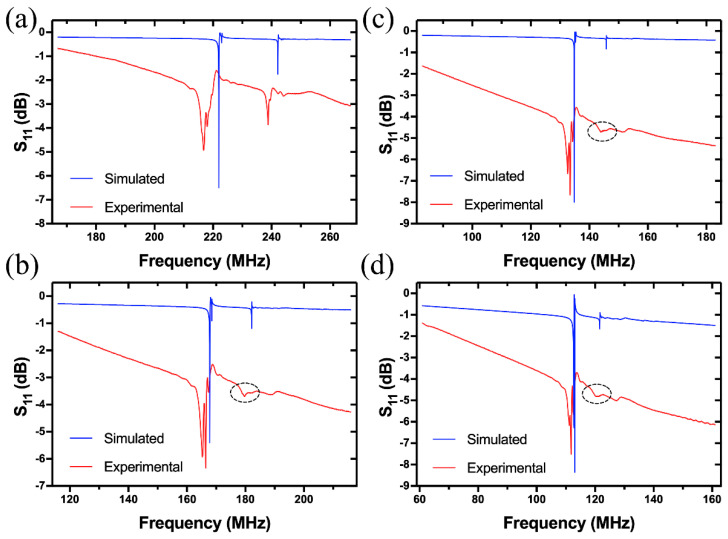
Comparison of the simulated and experimental reflection spectra of Device A when λ is 12 μm (**a**), 16 μm (**b**), 20 μm (**c**) and 24 μm (**d**).

Figure 6 is a comparison of the simulated and experimental reflection spectra when λ=12, 16, 20, 24 μm. Peaks 1, 2 and 3 are all observed in the simulated and experimental $S_{11}$ spectra for the four wavelengths, and the resonant frequencies agree well with each other generally. The resonance amplitude of Peak 2 is much weaker than Peak 1, which is also the case for λ=6.32 μm as shown in Figure 3a. Therefore, it is considered that Mode II and the lower edge of Mode I stopband are more valuable for practical applications. When λ=16, 20, 24 μm, Mode II is considerably weak even unrecognizable as marked by the circle in Figure 6. This may be because less acoustic energy of the SH-type leaky wave is confined on the surface of LGS substrate when $\frac{h}{\lambda }<1\%$ (i.e., λ>16 μm for h=160 nm), which is also manifested in Figure 4 and Figure 5.

The above analysis provides a guidance for the design of Pt/LGS SAW devices with (0°, 138.5°, 27°) cut. To precisely fabricate the device with required frequency, especially 433 MHz for wireless sensing, the velocity dispersion must be considered during device design. If the Pt/LGS SAW device structure is used for dual-mode sensing applications, it is better for h/λ to be larger than 1.3% (i.e., λ<12 μm when h=160 nm) for a strong resonant amplitude. As shown in Figure 3, the frequency interval between Mode I and II increases with the h/λ, which reaches about 40 MHz when λ=6.32 μm (frequency close to 433 MHz ISM band). This wide frequency interval helps to avoid bandwidth interference between antennas when Mode I and II are utilized for dual-mode sensing. Considering that Mode I and II are different types of acoustic waves, it can be expected that they have different sensitivities to environmental disturbances such as temperature, strain and pressure. Hence, for specific sensing purposes, it should be taken into account which mode is more suitable for the application.

As discussed above, the acoustic velocity dispersion is very significant, up to −131 m/s when h/λ increases from 0.67% to 2.53%. A reduced acoustic velocity means a higher requirement for the photolithography precision for finer patterns. An alternative strategy to suppress the velocity dispersion is to reduce the thickness (h) of Pt electrode. To investigate this, the thickness of Pt electrode is set to 160 and 100 nm respectively in the 3D model of Device A, and the velocities of the two modes are calculated and compared as a function of wavelength.

Figure 3c shows the effect of Pt electrode thickness on acoustic velocity. When h is decreased from 160 to 100 nm, the acoustic velocity dispersions for the two modes are apparently reduced. Quantitatively, the Δv of Mode I is reduced from −131 m/s to −59 m/s, and that of Mode II is reduced from −102 m/s to only −22 m/s, which is even ignorable. The modulation effect can be explained by that less acoustic energy is coupled into the Pt electrode for a smaller film thickness (h), thus the velocity slowing down effect of the mechanical resonator is reduced. The above analysis shows that the thickness of Pt film should be precisely controlled during device fabrication since it will significantly affect the resonant frequency of the device. It is also noteworthy that a thinner Pt film will cause the increase of electrode resistance, thus affecting the reflection characteristics of devices and deteriorating high temperature performance.

**Figure 7:**
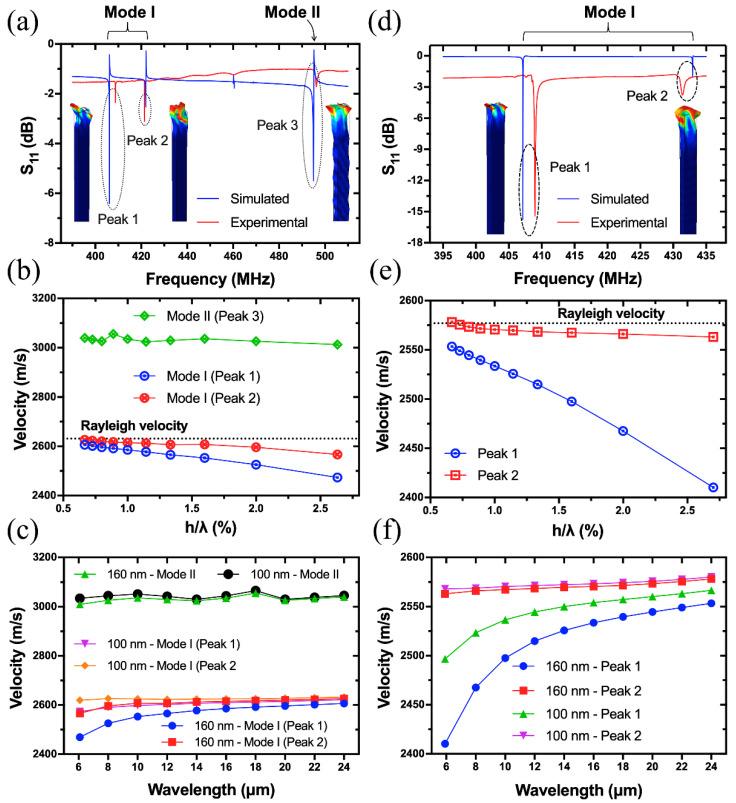
Comparison of the simulated and experimental $S_{11}$ spectra of Device B (λ=6.08 μm) (**a**); Dispersion relations between acoustic velocity and normalized Pt electrode thickness (h/λ) for the two modes of Device B (**b**); Modulation effect of Pt electrode thickness on acoustic velocities of Device B (**c**); Comparison of the simulated and experimental $S_{11}$ spectra of Device C (λ=5.92 μm) (**d**); Dispersion relationship between acoustic velocity and normalized Pt electrode thickness (h/λ) of Device C (**e**); Modulation effect of Pt electrode thickness on acoustic velocities of Device C (**f**).

The proposed 3D model will be used to study Device B and C in the following section. Similar results were obtained for Device B and C, and they are summarized in Figure 7. There are also two resonant modes (named as Mode I and II as above) in Device B, which are the Rayleigh-type SAW (Peak 1 and 2) and SH-type leaky wave (Peak 3) respectively as indicated by the $S_{11}$ spectra in Figure 7a. It is seen from Figure 7b that the velocities of Peak 1 and 2 exhibit strong dispersion relations with h/λ, while the velocity variation of Mode II is not obvious. This is because that Mode II almost vibrates in the whole body of LGS substrate, and thus the acoustic energy penetrated into Pt electrode is less affected by the change of wavelength as discussed above in detail. When the thickness of Pt electrode is decreased from 160 to 100 nm, the velocity dispersions of Peak 1 and 2 are suppressed obviously, and Peak 3 still keeps a low velocity dispersion in the wavelength range from 6.08 to 24 μm.

Figure 7d is a comparison of the experimental and simulated $S_{11}$ spectra of Device C (λ=5.92 μm). From the mode shape and energy confinement it can be concluded that both Peaks 1 and 2 are the Rayleigh modes as discussed above in detail. It is shown in Figure 7e that the velocity dispersion of Peak 1 is much higher than that of Peak 2. This is attributed to the relatively smaller penetration depth of Peak 1 into LGS substrate, which can also be observed from the mode shape in Figure 7d, and it was verified by a displacement analysis. Figure 7f shows the modulation effect of Pt electrode thickness on acoustic velocities of the two peaks.

From all the above results of devices A, B and C, the following conclusions can be made. Devices A and B have two resonant modes, namely the Rayleigh-type SAW and SH-type leaky waves, and the velocity of the latter is much higher than the former. Only one mode (Rayleigh-type SAW) is observed in Device C, which can be explained by the supposition that that the SH-type leaky wave is too weak to be measurable in the fabricated device because some spurious peaks were found in the higher frequency range during simulation. If a differential method is utilized, then the dual modes of devices A and B could be utilized for multi-parameter monitoring or temperature compensation in harsh environments such as gas turbines, where high temperature, high pressure and large strain (mostly on the turbine blade) often coexist and multi-mode sensing is required. The dispersion relation between acoustic velocity and h/λ indicates that both λ and h can be used to control the acoustic velocity, hence the resonant frequency of devices. Some previous works reported that, from room temperature upwards, LGS (0°, 138.5°, 27°) SAW device has a monotonous frequency-temperature characteristic, while LGS (0°, 138.5°, 117°) SAW device features a non-monotonic temperature characteristic, with a relatively larger strain sensitivity than the former [10,11,36]. However, these results all refer to the Rayleigh mode of the LGS SAW devices. That is to say, the sensing characteristics of the SH-type leaky wave are yet to be explored, that are expected to be different from those of the Rayleigh-type SAW. Investigation on this will be considered in our future work.

### 4.2. Effect of Al_2_O_3_ Passivation Layer on Wave Modes

**Figure 8:**
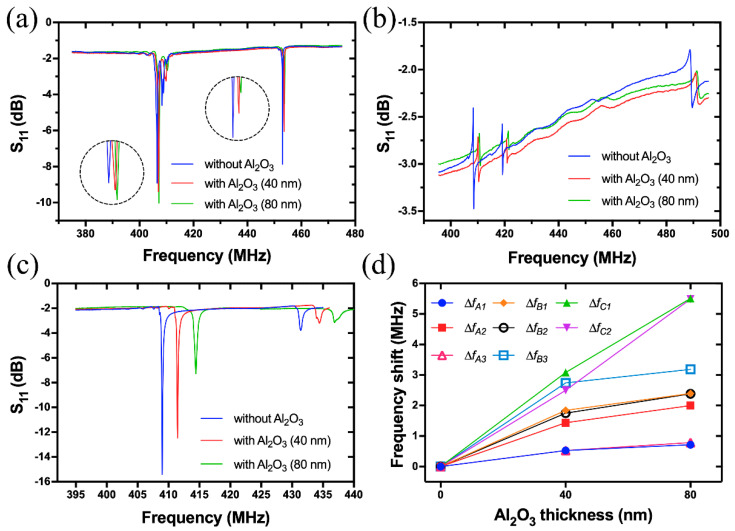
Effects of Al_2_O_3_ passivation layer with different thicknesses on the reflection spectra of Device A (**a**), Device B (**b**) and Device C (**c**); Frequency shift of each resonant peak as a function of Al_2_O_3_ layer thickness (**d**).

To improve the stability and life-time of Pt/LGS SAW devices in high temperature environment, an Al_2_O_3_ passivation layer is commonly required. Hence, it is necessary to investigate effects of the Al_2_O_3_ passivation on wave modes of Pt/LGS SAW devices. In this work, an Al_2_O_3_ layer with a thickness of 40 and 80 nm was deposited by atomic layer deposition (ALD) on the top of devices A, B and C, and the reflection spectra were measured and compared. The results in Figure 8a–c show that the wave modes and resonant peaks of devices A, B and C remain the same even after the deposition of an Al_2_O_3_ layer.

However, the resonant frequencies are increased with the increase of Al_2_O_3_ thickness, and $\Delta f_{c}>\Delta f_{B}>\Delta f_{A}$, where $\Delta f_{A}$, $\Delta f_{B}$ and $\Delta f_{c}$ are respectively the frequency shift of devices A, B and C after the deposition of an Al_2_O_3_ layer. Figure 8d summarizes the frequency shifts of each peak of devices A, B and C. It is shown that with an 80 nm Al_2_O_3_ layer, Peak 1 of Device C has the maximum frequency shift ($\Delta f_{c1}$) of 5.51 MHz, and Peak 1 of Device A has the minimum value ($\Delta f_{A1}$) of 0.71 MHz. This frequency up-shift phenomenon is attributed to the larger acoustic velocity of Al_2_O_3_ ($v_{\mathrm{longitudinal}}=9900\;m/s$, $v_{\mathrm{shear}}=5800\;m/s$) compared to those of LGS, which are 2743, 2631 and 2577 m/s for Euler angles of (0°, 138.5°, 27°), (0°, 138.5°, 117°) and (0°, 138.5°, 72°), respectively. Obviously, the velocity difference between LGS (0°, 138.5°, 72°) and Al_2_O_3_ is the largest, which causes the remarkable velocity enhancement introduced by the Al_2_O_3_ passivation layer. However, it can also be seen that an additional Al_2_O_3_ layer deteriorates resonance amplitude for all the devices. Therefore, the thickness of Al_2_O_3_ passivation layer should be seriously considered during the design of Al_2_O_3_/Pt/LGS SAW device.

## 5. Conclusions

This work presented a detailed mode analysis for the Pt/LGS SAW devices both experimentally and theoretically. Three commonly used LGS cuts with Euler angles of (0°, 138.5°, 27°), (0°, 138.5°, 117°) and (0°, 138.5°, 72°) were considered, which respectively correspond to devices A, B and C. The fabrication steps of the devices were described, where AZ5214E image reversal resist was used to improve the lift-off process. By displacement analysis, it was found that there is a large SH displacement component in the devices, and thus 3D COMSOL simulation was adopted to model the devices. Both experimental and simulated results indicated that there are two modes (three resonant peaks) excited in devices A and B, which are Rayleigh-type SAW and SH-type leaky wave respectively. Only the Rayleigh-type mode (two resonant peaks) was observed in the reflection spectrum ($S_{11}$) of Device C. For all the three devices, a dispersion relation between acoustic velocity and h/λ was obtained, and the dispersion of Rayleigh-type mode was more significant. The modulation effect of Pt film thickness on velocity dispersion was investigated, and it was found that a thinner Pt film can help to suppress velocity dispersion. It also revealed that the Al_2_O_3_ passivation layer has no effect on the wave mode, but can increase the resonant frequencies, which is attributed to the high acoustic velocity of the Al_2_O_3_ layer. In conclusion, this work provides a better understanding of the acoustic modes of Pt/LGS SAW devices, as well as guidance for device design, especially referring to the parameters such as LGS cut, acoustic velocity, wavelength, electrode thickness, and Al_2_O_3_ thickness. It is believed that the Rayleigh-type SAW and SH-type leaky wave sensors have great potential for dual-mode sensing applications in hostile environment, to achieve multi-parameter monitoring or temperature-compensation on a single chip.

## Figures and Tables

**Table 1:** Summary of calculated parameters of LGS using different methods.

Euler Angle	Numerical Computation		2D Simulation		3D Simulation		Maximum SH Disp.(nm)
	$v_{f}$ (m/s)	$v_{m}$ (m/s)	$v_{f}$ (m/s)	$v_{m}$ (m/s)	$v_{f}$ (m/s)	$v_{m}$ (m/s)	
(0°, 138.5°, 27°)	2743.0	2738.4	2815.9	2814.5	2755.7	2750.5	2.5
(0°, 138.5°, 117°)	2631.0	2630.6	2889.2	2888.8	2638.2	2637.8	14
(0°, 138.5°, 72°)	2577.0	2575.4	2919.7	2919.5	2577.2	2575.7	6
